# Incorporating early and late-arriving photons to improve the reconstruction of cerebral hemodynamic responses acquired by time-resolved near-infrared spectroscopy

**DOI:** 10.1117/1.JBO.26.5.056003

**Published:** 2021-05-17

**Authors:** Daniel Milej, Androu Abdalmalak, Ajay Rajaram, Amandeep Jhajj, Adrian M. Owen, Keith St. Lawrence

**Affiliations:** a Lawson Health Research Institute, Imaging Program, London, Ontario, Canada; b Western University, Department of Medical Biophysics, London, Ontario, Canada; c Western University, Brain and Mind Institute, London, Ontario, Canada

**Keywords:** diffuse reflectance, near-infrared spectroscopy, time-resolved measurements, hypercapnia, functional activation, brain imaging

## Abstract

**Significance:** Despite its advantages in terms of safety, low cost, and portability, functional near-infrared spectroscopy applications can be challenging due to substantial signal contamination from hemodynamics in the extracerebral layer (ECL). Time-resolved near-infrared spectroscopy (tr NIRS) can improve sensitivity to brain activity but contamination from the ECL remains an issue. This study demonstrates how brain signal isolation can be further improved by applying regression analysis to tr data acquired at a single source–detector distance.

**Aim:** To investigate if regression analysis can be applied to single-channel trNIRS data to further isolate the brain and reduce signal contamination from the ECL.

**Approach:** Appropriate regressors for trNIRS were selected based on simulations, and performance was evaluated by applying the regression technique to oxygenation responses recording during hypercapnia and functional activation.

**Results:** Compared to current methods of enhancing depth sensitivity for trNIRS (i.e., higher statistical moments and late gates), incorporating regression analysis using a signal sensitive to the ECL significantly improved the extraction of cerebral oxygenation signals. In addition, this study demonstrated that regression could be applied to trNIRS data from a single detector using the early arriving photons to capture hemodynamic changes in the ECL.

**Conclusion:** Applying regression analysis to trNIRS metrics with different depth sensitivities improves the characterization of cerebral oxygenation signals.

## 1 Introduction

The interest in near-infrared spectroscopy (NIRS) for mapping regional brain activation associated with functional tasks is growing rapidly, as reflected by the increasing number of commercial systems.^1^^–^^3^ Despite advantages in terms of safety, low cost, and portability, functional near-infrared spectroscopy (fNIRS) applications using continuous-wave (CW) technologies are limited in insolating signals from the brain. Substantial signal contamination can result from changes in hemodynamics in the extracerebral layer (ECL).^4^^,^^5^ As a consequence, a number of techniques have been proposed to reduce the influence of ECL signal contributions. An increasingly popular approach is to collect light intensities at a short source–detector separation (i.e., $r_{\mathrm{SD}}∼1\;\mathrm{cm}$), which is predominately sensitive to scalp hemodynamics while simultaneously collecting data at a larger separation ($r_{\mathrm{SD}}\geq 3\;\mathrm{cm}$).^6^ Assuming the two signals contain the same ECL contributions, the short separation signal can be used as a regressor to filter the ECL interference from the main signal, improving the quality of the recovered brain signal.^6^^–^^8^

An alternative strategy is to enhance the depth sensitivity of the measurements using time-resolved NIRS (trNIRS), which uses picosecond light pulses and fast detectors to record the distribution of times-of-flight (DTOF) of diffusely reflected photons.^9^ As DTOFs contain both time and intensity information, absorption changes at different depths can be resolved since photon arrival times are proportional to path lengths. The most popular depth-enhancing methods are based on calculating the statistical moments of a DTOF^10^^,^^11^ or integrating the photon counts within time windows/gates.^12^^,^^13^ In both cases, the goal is to focus on late-arriving photons since they have the greatest probability of interrogating the brain. Previous studies using layered tissue-mimicking phantoms, animal models, and human subjects have shown that trNIRS provides superior sensitivity to cerebral hemodynamics compared to conventional CW NIRS.^13^^–^^17^ Moreover, studies conducted on healthy subjects and patients have shown that signals extracted using either approach provide superior correlation with hemodynamic changes in the brain.^9^^,^^18^

Despite the enhanced depth sensitivity of trNIRS, signals weighted toward late-arriving photons can still be influenced by hemodynamic fluctuations in the ECL.^19^ This was demonstrated in a recent trNIRS study focused on measuring cerebrovascular reactivity in response to a global vascular stimulus (hypercapnia).^20^ Contamination from the scalp was evident by distortions in the oxy- and deoxyhemoglobin signals recorded at $r_{\mathrm{SD}}=3$ and 4 cm that mirrored the recordings at $r_{\mathrm{SD}}=1\;\mathrm{cm}$. By applying higher moment analysis, these effects were substantially diminished but not completely removed.^20^ Alternatively depth sensitivity can be further enhanced by subtracting higher moments of DTOFs recorded at two source–detector distances;^21^ however, the application of this approach is challenging due to the substantial reduction in contrast-to-noise ratio compared to analyzing individual DTOFs.^22^

In this study, an alternative technique was investigated that combines trNIRS with a regression approach analogous to short source-detector measurements used in CW fNIRS studies.^6^^,^^7^ Unlike CW NIRS that requires an additional channel to act as the regressors, regression can be applied to trNIRS data from a single detector by utilizing the depth information provided in the recorded DTOFs. In this application, a signal that is predominately weighted by the ECL can be extracted from a DTOF by focusing on early arriving photons and subsequently used as a regressor to remove ECL interference from signals with greater depth sensitivity, such as those obtained from higher moments or later gates. An additional advantage of trNIRS is that both the regressor and the dependent variable are extracted from data collected by the same detector. This avoids potential artifacts that can arise when the two signals are recorded by sensors located at different locations, considering the regression approach is based on the assumption that the regressor and dependent variable contain the same physiological nuisance signal. It has been demonstrated that the correlation between physiological signals recorded using separate sensors diminishes with an increasing distance between each sensor due to time delays and spatial variations in the hemodynamic properties of superficial tissue.^8^^,^^23^

The ability to improve the isolation of the brain signal by adapting regression analysis to trNIRS was evaluated using data from two previous studies using trNIRS to measure oxygenation responses to hypercapnia^20^ and functional activation.^24^ The former was chosen because the rapid and relatively large increase in end-tidal carbon dioxide pressure (∼15  mmHg) elicited a substantial systemic effect, as indicated by a significant change in arterial blood pressure, in addition to the expected cerebral response. Differences in ECL and cerebral contributions were evident by comparing signals recorded at $r_{\mathrm{SD}}=1$ and 3 cm. Consequently, this data set was used to evaluate the ability of the regression approach to remove ECL contributions when applied to DTOFs recorded at $r_{\mathrm{SD}}=3\;\mathrm{cm}$. The functional study^24^ involved a motor imagery task that invoked activation in motor planning regions and was included to demonstrate the feasibility of applying the regression approach to functional trNIRS data. Both applications involved two common approaches used to extract depth information from DTOFs, time gating and statistical moment analysis.^10^^,^^13^ In addition to these applications, this study includes a sensitivity analysis conducted to select the appropriate trNIRS metrics for regression analysis.

## 2 Methods

All experiments were approved by the Western University Health Sciences Research Ethics Board, which adheres to the guidelines of the Tri-Council Policy Statement for research involving humans. Written informed consent was obtained from each subject prior to the experiment. All subjects had no history of neurological or psychiatric disorders.

### 2.1 Instrumentation

All data were collected using an in-house built trNIRS system.^25^^–^^27^ The device included two pulsed laser heads emitting at λ=760 and 830 nm, controlled by a Sepia II laser driver operating at 80 MHz (PicoQuant, Germany). The laser heads were coupled to multimode bifurcated fiber [φ=0.4  mm, numerical aperture (NA)=0.39, Thorlabs] to deliver the light to the scalp. A set of four detection fiber bundles (φ=3.6  mm, NA=0.55, Fiberoptics Technology) collected diffusively reflected light from the scalp. Each bundle was coupled to a hybrid photomultiplier tube (PMA Hybrid 50, PicoQuant, Germany).^28^ A time-correlated single-photon counting module (HydraHarp 400, PicoQuant, Germany) was used to generate the photon arrival times and DTOFs were generated by in-house software written in LabVIEW (National Instruments).^21^

All data collected in the hypercapnia and motor imagery protocols described in Secs. 2.2 and 2.3 were acquired continuously at a sampling rate of 3.33 Hz. At the end of every study, the instrument response function (IRF) was measured using a custom-built light-tight box that connected the emission fiber to a detection probe with a separation of 6 cm. On average, the full width at half maximum was 0.579±0.005  ns at 760 nm and 0.653±0.006  ns at 830 nm.

### 2.2 Experimental Protocol: Hypercapnia

**Fig. 1:**
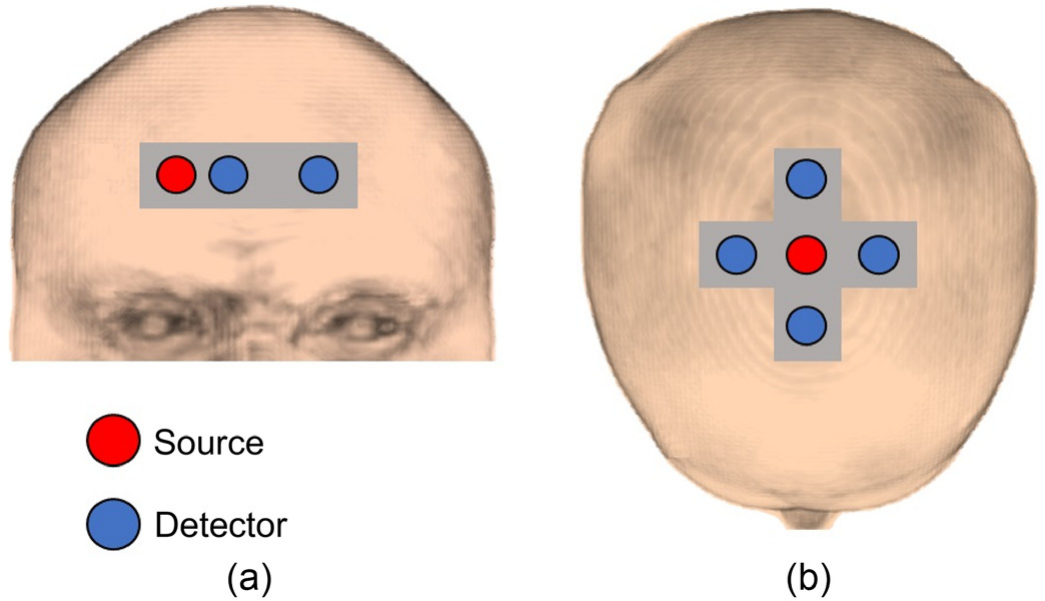
Schematic of the design and location of the probe holders used in (a) the hypercapnia protocol and (b) the functional activation protocol.

Data were obtained from a previous study that involved 11 healthy participants (three females, eight males, aged 25 to 36 y, mean=28±3 y).^20^ Subjects sat on a reclining chair while wearing a facemask connected to a computer-controlled gas delivery circuit (RespirAct™, Thornhill Research Inc, Toronto, Canada). A custom-designed probe holder was placed on the subject’s forehead and secured by a velcro headband. Two detection fiber bundles were placed at $r_{\mathrm{SD}}=1$ and 3 cm [Fig. 1(a)]. The experimental protocol consisted of three 2 min periods of hypercapnia in which end-tidal carbon dioxide pressure ($P_{\mathrm{ET}}{\mathrm{CO}}_{2}$) was increased by 15 mmHg above each subject’s normocapnic $P_{\mathrm{ET}}{\mathrm{CO}}_{2}$ value, as determined by the gas delivery circuit. The first period started two minutes after baseline recordings and each hypercapnia period was followed by 5 min of normocapnia.

### 2.3 Experimental Protocol: Functional Activation

Data were extracted from a previous study involving 11 healthy participants (three females, eight males, aged 24 to 40 y, mean=28±4 y).^24^ With each participant seated, trNIRS probes were positioned on the head to detect activation in the motor planning regions (supplementary motor area and the premotor cortex). The emission fiber was centred over FCz, according to the international system for electroencephalography, and the detection fibers bundles were secured in a cross pattern at a source-detector separation of 3 cm [Fig. 1(b)]. The activation paradigm consisted of a 30-s baseline period followed by five 30-s sequential blocks of motor imagery for a total acquisition time of 5.5 min. During the task periods, participants were asked to remain as still as possible and imagine hitting a tennis ball repeatedly as if they were playing a vigorous game of tennis.

### 2.4 Time-Resolved Depth Sensitivity—Theory

Two approaches for extracting depth information from DTOFs are by time gating and calculating the first three statistical moments: the zeroth moment (total number of photons, N), the first moment (mean time of flight, ⟨t⟩), and the second central moment (variance, V). Higher moments are more sensitive to late-arriving photons due to the positive skewness of the DTOF.^10^^,^^11^ With the former approach, each DTOF is divided into time gates, and the photon count is integrated within each gate.^12^^,^^29^ Similar to higher moments, gates positioned on the tail of a DTOF provide the greatest depth sensitivity, whereas the gates located on the left side of the DTOF are more sensitive to more superficial layers. Based on these considerations, combinations of statistical moments and time gates can be used to extract signals with different depth sensitivities from a single DTOF.

**Fig. 2:**
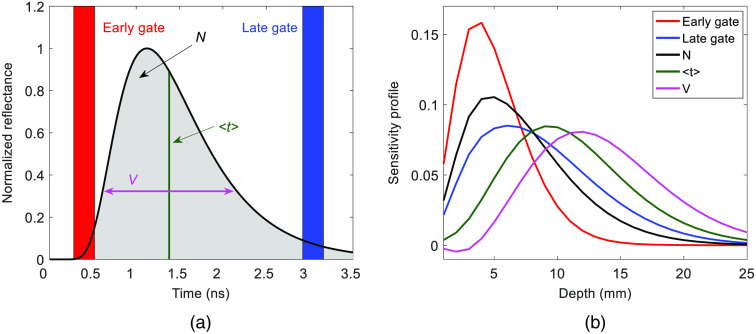
(a) Theoretical DTOF generated for $r_{\mathrm{SD}}=3\;\mathrm{cm}$. Overlaid on the DTOF is the locations of early and late gates (width=250  ps) and the first three statistical moments. (b) Corresponding sensitivity profiles for the two gates and the three statistical moments generated using $\mu_{a}=0.1\;{\mathrm{cm}}^{-1}$ and ${\mu_{s}}^{\prime }=10\;{\mathrm{cm}}^{-1}$.

Figure 2 shows an example of a theoretical DTOF and the corresponding sensitivity profiles of the signals extracted from various gates and moments. The DTOF was generated using the analytical solution to the diffusion approximation for a semi-infinite homogeneous medium for a source-detector separation of 3 cm, an absorption coefficient ($\mu_{a}$) of $0.1\;{\mathrm{cm}}^{-1}$, and a reduced scattering coefficient (${\mu_{s}}^{\prime }$) of $10\;{\mathrm{cm}}^{-1}$.^30^ The sensitivity profiles were also generated from the solution to the diffusion approximation for a semi-infinite homogeneous medium following the approach outlined by Kacprzak et al.^31^ Briefly, the medium was divided into a grid of $0.1\times 0.1\times 0.1\;{\mathrm{cm}}^{3}$ voxels and the signal response to a change in $\mu_{a}$ of $0.01\;{\mathrm{cm}}^{-1}$ was recorded within each consecutive voxel. Next, the sensitivity factors for consecutive layers (each with a thickness of 1 mm) were generated by summing the sensitivity values for all voxels within a layer. The same optical properties used to generate the theoretical DTOF were used to define the background optical properties for calculating the sensitivity profiles.^15^^,^^21^^,^^32^ Figure 2(a) shows the three statistical moments, as well as early and late gates (gate width=250  ps). The sensitivity profiles for these two gates and the three moments are shown in Fig. 2(b). Observable differences in these sensitivity profiles indicate that signals weighted differently by the ECL and brain can be extracted from data recorded using a single source–detector separation.

### 2.5 Analysis of Recorded DTOFs

**Fig. 3:**
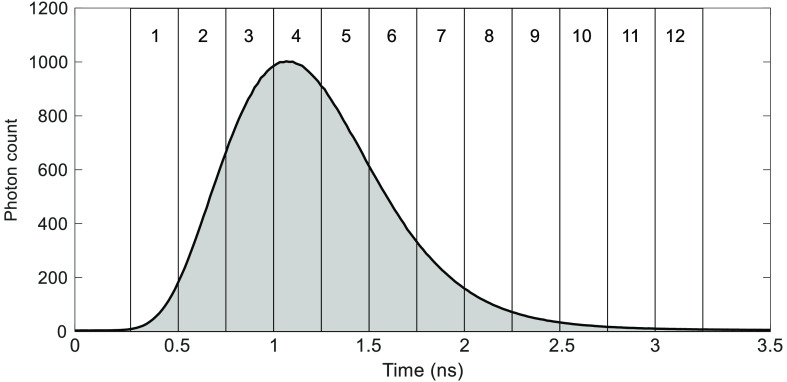
Position of 12 time gates (width=250  ps) used to extract attenuation signals from the DTOF. In this case, the DTOF was averaged over the 2 min baseline period for one subject from the hypercapnia study (λ=760  nm and $r_{\mathrm{SD}}=3\;\mathrm{cm}$).

For each time series of DTOFs recorded at 760 and 830 nm, the first three statistical moments (N, ⟨t⟩, and V) were calculated by setting the lower and upper integration limits based on arrival times corresponding to 1% of the peak of the DTOF. The change in each moment relative to its initial value was calculated to generate three time series (i.e., ΔN, Δ⟨t⟩, and ΔV) for the two wavelengths individually. Next, each DTOF was divided into 12 consecutive gates with a fixed width of 250 ps^29^ and the time-varying change in attenuation (ΔA) was calculated for each gate (Fig. 3). Similar to moment analysis, the start of the first gate was positioned at the rising edge of the DTOF when the signal intensity reached 1% of the peak value.

Time courses determined at 760 and 830 nm from the statistical moments and time gates were converted into the corresponding absorption changes $\Delta \mu_{a}(\lambda )$ using the sensitivity analysis described in Sec. 2.4. To improve the accuracy of the sensitivity profiles, an IRF (described in Sec. 2.1) was incorporated into the sensitivity profile calculations. The sensitivity factors were generated using average optical properties for the subjects in the hypercapnia study (760 nm: $\mu_{a}=0.14\pm 0.02\;{\mathrm{cm}}^{-1}$, ${\mu_{s}}^{\prime }=9.4\pm 1.2\;{\mathrm{cm}}^{-1}$; 830 nm: $\mu_{a}=0.13\pm 0.02\;{\mathrm{cm}}^{-1}$, and ${\mu_{s}}^{\prime }=8.1\pm 1.2\;{\mathrm{cm}}^{-1}$):^20^

$$\Delta \mu_{a}(\lambda )=\frac{\Delta S}{SF_{S}},$$ (1)

where ΔS represents the signal change for each of the statistical moments (ΔN, Δ⟨t⟩, and ΔV) and time gates (ΔA), and ${\mathrm{SF}}_{S}$ represents the corresponding sensitivity factor. The $\Delta \mu_{a}$ time courses were then converted to change in concentration of oxyhemoglobin ($\Delta C_{\mathrm{HbO}}$) and deoxyhemoglobin ($\Delta C_{\mathrm{Hb}}$):

$$\Delta \mu_{a}(\lambda )=\ln(10)\cdot (\varepsilon_{\mathrm{HbO}}(\lambda )\cdot \Delta C_{\mathrm{HbO}}+\varepsilon_{\mathrm{Hb}}(\lambda )\cdot \Delta C_{\mathrm{Hb}}),$$ (2)

where $\varepsilon_{\mathrm{HbO}}(\lambda )$ and $\varepsilon_{\mathrm{Hb}}(\lambda )$ are the molar extinction coefficients for oxy- and deoxyhemoglobin, respectively.

### 2.6 Regression Analysis

**Fig. 4:**
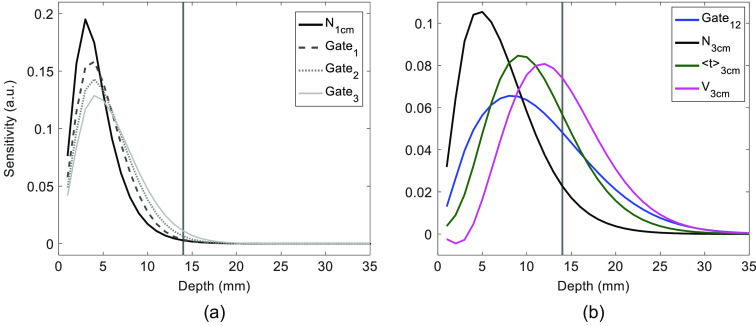
(a) Sensitivity profiles of the signals used as the regressor (i.e., N measured at $r_{\mathrm{SD}}=1\;\mathrm{cm}$ and the first three gates measured at $r_{\mathrm{SD}}=3\;\mathrm{cm}$). (b) Sensitivity profiles of the dependent signals measured at $r_{\mathrm{SD}}=3\;\mathrm{cm}$, which included the three statistical moments and the last gate (gate 12 for the hypercapnia data). The vertical gray line represents the average distance to the brain d=14  mm.

Regression analysis was based on the method proposed by Saager et al.,^6^ which was developed to isolate absorption trends in the lower layer of a two-layer turbid medium. The signal change in the lower layer (i.e., brain), $\Delta S_{D}$, was calculated according to

$$\Delta S_{D}=\Delta S_{F}-\alpha_{NF}\cdot \Delta S_{N},$$ (3)

where $\Delta S_{F}$ is the signal change for a parameter with greater depth sensitivity, $\Delta S_{N}$ is the signal change for a parameter primarily sensitive to the ECL, and $\alpha_{\mathrm{NF}}$ is the scaling parameter obtained by fitting $\Delta S_{N}$ to $\Delta S_{F}$ using a least-squares criterion. For the hypercapnia data sets, regression was first applied using the ΔN time series measured at $r_{\mathrm{SD}}=1\;\mathrm{cm}$ as the regressor ($\Delta N_{1\;\mathrm{cm}}$), analogous to original approach proposed for CW NIRS data. Next, $\Delta S_{N}$ was extracted from DTOFs measured at $r_{\mathrm{SD}}=3\;\mathrm{cm}$ based on the criterion that the selected signal had >97% sensitivity to the ECL. This value was based on an average distance from scalp to brain of 14 mm as measured by magnetic resonance imaging.^20^ Signals that satisfied this criterion were ΔA for gates 1 to 3 [Fig. 4(a)]. The dependent signals ($\Delta S_{F}$) used in Eq. (3) were obtained for DTOFs measured at $r_{\mathrm{SD}}=3\;\mathrm{cm}$ and included the three statistical moments and the late gate [Fig. 4(b)]. The last gate was the twelfth for the hypercapnia data and the tenth for the activation data.

### 2.7 Assessment of the Cerebrovascular Reactivity

For the hypercapnic challenge, the $\Delta C_{\mathrm{HbO}}$ and $\Delta C_{\mathrm{Hb}}$ signals calculated from individual moments and gates, as well as after applying regression analysis, were modelled as the convolution of the recorded change in $\Delta P_{\mathrm{ET}}{\mathrm{CO}}_{2}(t)$ and a hemodynamic response function (HRF):^20^

$$\Delta S(t)=ssCVR\cdot [\Delta P_{\mathrm{ET}}{\mathrm{CO}}_{2}(t)*(\frac{1}{P}e^{-\frac{\left(t-t_{0}\right)}{\tau }})],$$ (4)

where ΔS(t) is the signal change, ssCVR is the steady-state value of cerebrovascular reactivity (CVR), ∗ denotes the convolution operator, τ is the time constant defining the dynamic component of CVR, and $t_{0}$ is the time delay between $\Delta P_{\mathrm{ET}}{\mathrm{CO}}_{2}(t)$ and ΔS(t). The remaining term in the convolution is the HRF, which has units of 1/time with $P=\int_{0}^{\infty }e^{-t/\tau }dt$. Best-fit estimates of τ, $t_{0}$ and ssCVR were obtained by numerical optimization (fminsearchbnd, MATLAB, Mathworks Inc.). The fitting was performed over a time window that started at the beginning of the 2 min hypercapnia period and encompassed the 5 min recovery period following hypercapnia. It is worth noting, in this application ssCVR has units of μM/mmHg, reflecting the fact that reactivity is being characterized in terms of change in hemoglobin concentration, as opposed to the conventional definition of change in cerebral blood flow per mmHg.

### 2.8 Functional Data Analysis

The functional data were analyzed using the same approach described in Secs. 2.5 and 2.6, except the average of ΔA signals from gates 2 and 3 were used as the regressor to improve the signal-to-noise ratio, and the late gate was number 10 instead of 12, which was used when analyzing the hypercapnia data. It was necessary to stop at gate 10 due to lower photon counts observed in the functional activation data. Next, analysis based on the general linear model (GLM) using the canonical HRF was performed for hemoglobin signals calculated using gate 10, the variance, and the signals obtained following regression analysis. Finally, to assess the improvement in reconstructed HRF, a chi-square ($\chi^{2}$) goodness of fit was calculated for signals prior to and after applying regression.

### 2.9 Statistical Analysis

All data are presented as mean ± standard deviation unless otherwise noted. Statistical analyses were conducted in MATLAB (using Statistics and Machine Learning Toolbox). Statistical significance was defined as p<0.05. An unequal variance t-test was used to investigate differences in fitting parameters (τ and *ssCVR*) before and after applying regression with $\Delta N_{1\;\mathrm{cm}}$ as the regressor. This was performed for each $\Delta C_{\mathrm{HbO}}$ and $\Delta C_{\mathrm{Hb}}$ time series derived from the three statistical moments measured at $r_{\mathrm{SD}}=3\;\mathrm{cm}$ ($\Delta N_{3\;\mathrm{cm}}$, $\Delta {\left\langle t\right\rangle }_{3\;\mathrm{cm}}$, $\Delta V_{3\;\mathrm{cm}}$). Similarly, a t-test was used to investigate differences in τ and *ssCVR* before and after applying regression with an early gate signal ($r_{\mathrm{SD}}=3\;\mathrm{cm}$) as the regressor. This analysis was performed for $\Delta C_{\mathrm{HbO}}$ and $\Delta C_{\mathrm{Hb}}$ time series derived from gate 12 ($r_{\mathrm{SD}}=3\;\mathrm{cm}$) and $\Delta V_{3\;\mathrm{cm}}$. A one-way analysis of variance (ANOVA) was used to investigate if τ and *ssCVR* estimates changed depending on which early gate (1 to 3) was used as the regressor. A t-test was used to investigate differences in the τ and *ssCVR* estimates derived using $\Delta N_{1\;\mathrm{cm}}$ or an early gate signal recorded at $r_{\mathrm{SD}}=3\;\mathrm{cm}$ as the regressor. Finally, a t-test was used to investigate differences in $\chi^{2}$ goodness of fit before and after applying regression to activation data. In this application, the early gate signal recorded at $r_{\mathrm{SD}}=3\;\mathrm{cm}$ was used as the regressor.

## 3 Results

**Fig. 5:**
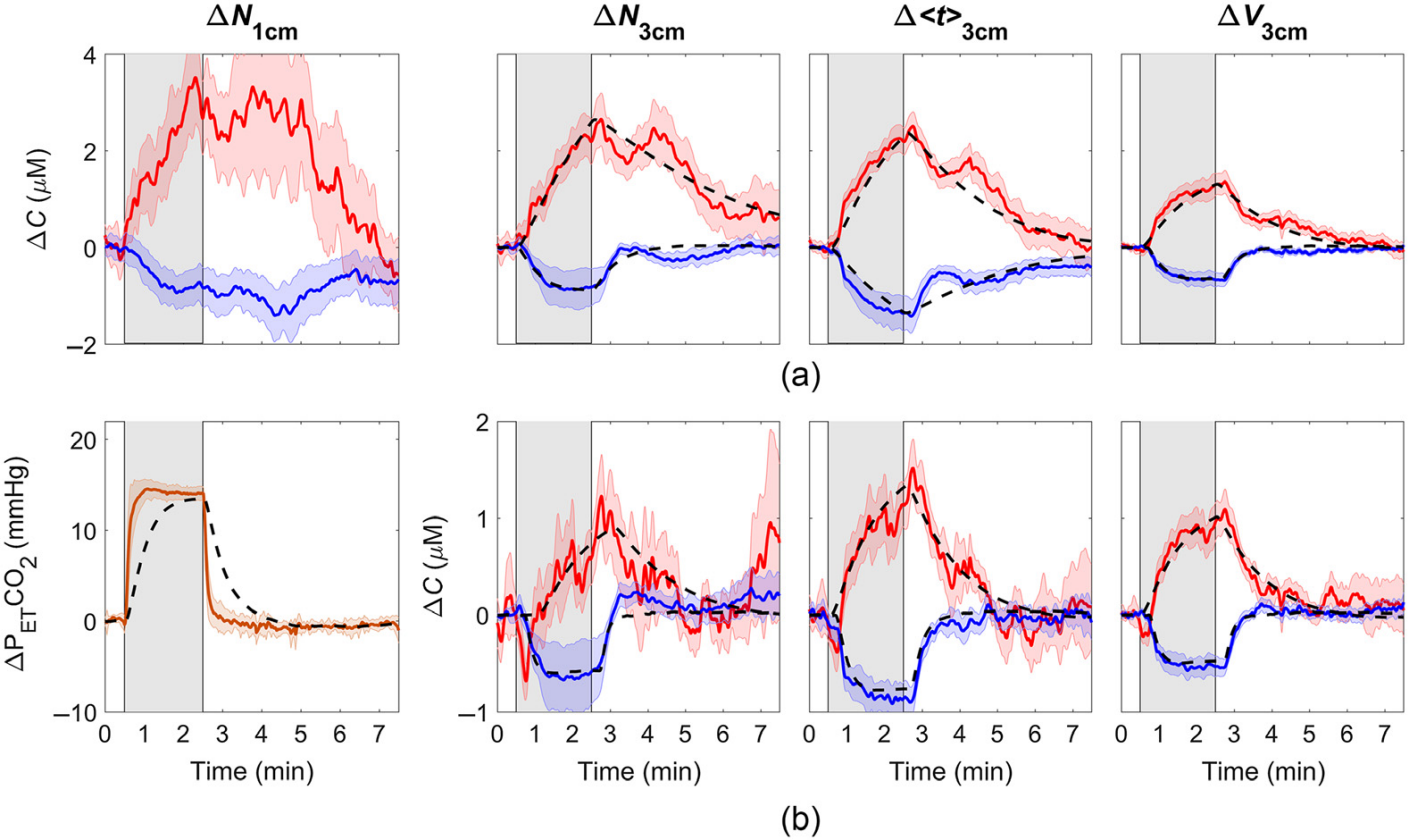
(a) Average $\Delta C_{\mathrm{HbO}}$ (red) and $\Delta C_{\mathrm{Hb}}$ (blue) responses to a 2 min hypercapnic challenge (indicated by the gray shaded region). Time courses are presented for the signals measured at two source-detector separations: $r_{\mathrm{SD}}=1$ and 3 cm and were averaged across nine subjects. The best fit of the hemodynamic model to each averaged hemoglobin time course is illustrated by the black dashed line. (b) Corresponding hemoglobin signals obtained after regressing the $\Delta N_{1\;\mathrm{cm}}$ from the time courses measured at $r_{\mathrm{SD}}=3\;\mathrm{cm}$ for the three moments. Average recorded $\Delta P_{\mathrm{ET}}{\mathrm{CO}}_{2}$ is presented in the left column, along with a theoretical ΔS(t) generated for τ=27  s.

Figure 5(a) displays the average $\Delta C_{\mathrm{HbO}}$ and $\Delta C_{\mathrm{Hb}}$ responses to the 2 min hypercapnia challenge. Data are presented for both source-detector separations and each of the three moments. These time courses illustrate how the dynamics of the hypercapnic response varied with $r_{\mathrm{SD}}$ and statistical moments. Included in each figure is the best fit of the hemodynamic model described in Sec. 2.7. Figure 5(b) shows the $\Delta C_{\mathrm{HbO}}$ and $\Delta C_{\mathrm{Hb}}$ time courses obtained from the regression analysis using $\Delta N_{1\;\mathrm{cm}}$ as the regressor. These time courses show that the dynamics of the reconstructed hypercapnic response were improved for all three statistical moments, as demonstrated by the removal of the substantial residue signals that were observed after $P_{\mathrm{ET}}{\mathrm{CO}}_{2}$ had returned to normocapnia. The first graph in Fig. 5(b) shows the average $\Delta P_{\mathrm{ET}}{\mathrm{CO}}_{2}$ time course, which illustrates the highly reproducible hypercapnic response across subjects, and the corresponding ΔS(t) generated from Eq. (4) using τ=27  s, which was obtained from previous BOLD studies of dynamic CVR.^33^^,^^34^ The theoretical bold signal is provided to indicate the signal expected from NIRS if there was no contamination from the ECL.

Table 1 provides averaged best-fit values of τ and ssCVR, from the analysis of $\Delta C_{\mathrm{HbO}}$ and $\Delta C_{\mathrm{Hb}}$ time series, derived for the individual moments measured at $r_{\mathrm{SD}}=3\;\mathrm{cm}$ [Fig. 5(a)] and following regression using $\Delta N_{1\;\mathrm{cm}}$ as the regressor [Fig. 5(b)]. The mean values of τ did not include two outliers that exceeded the upper boundary of the fitting range τ=250  s (same subjects for both $\Delta C_{\mathrm{HbO}}$ and $\Delta C_{\mathrm{Hb}}$).

**Table 1:** Best fit estimates of the time constant (τ) and steady-state cerebrovascular reactivity (ssCVR) obtained from the analysis of the $\Delta C_{\mathrm{HbO}}$, $\Delta C_{\mathrm{Hb}}$ data derived from the three moments recorded at $r_{\mathrm{SD}}=3\;\mathrm{cm}$ and the regression approach applied to each moment separately. Values are presented as average (standard deviation) across subjects.

		Regressor	τ (s)			ssCVR (μM/mmHg)		
Oxyhemoglobin (HbO)	Moment analysis	—	$\Delta N_{3\;\mathrm{cm}}$	$\Delta {\left\langle t\right\rangle }_{3\;\mathrm{cm}}$	$\Delta V_{3\;\mathrm{cm}}$	$\Delta N_{3\;\mathrm{cm}}$	$\Delta {\left\langle t\right\rangle }_{3\;\mathrm{cm}}$	$\Delta V_{3\;\mathrm{cm}}$
			72±42	82±45	77±45	0.18±0.05	0.15±0.05	0.09±0.02
	Regression approach	$\Delta N_{1\;\mathrm{cm}}$	53±14	48±43^a^	43±40^a^	0.07±0.07^a^	0.07±0.07^a^	0.06±0.03^a^
Deoxyhemoglobin (Hb)	Moment analysis	—	$\Delta N_{3\;\mathrm{cm}}$	$\Delta {\left\langle t\right\rangle }_{3\;\mathrm{cm}}$	$\Delta V_{3\;\mathrm{cm}}$	$\Delta N_{3\;\mathrm{cm}}$	$\Delta {\left\langle t\right\rangle }_{3\;\mathrm{cm}}$	$\Delta V_{3\;\mathrm{cm}}$
			64±27	62±31	31±13	−0.08±0.07	−0.10±0.06	−0.05±0.03
	Regression approach	$\Delta N_{1\;\mathrm{cm}}$	26±32^a^	32±28	21±24	−0.05±0.06	−0.06±0.04	−0.04±0.02

a Statistically significant differences between parameters recovered with and without regression analysis

**Fig. 6:**
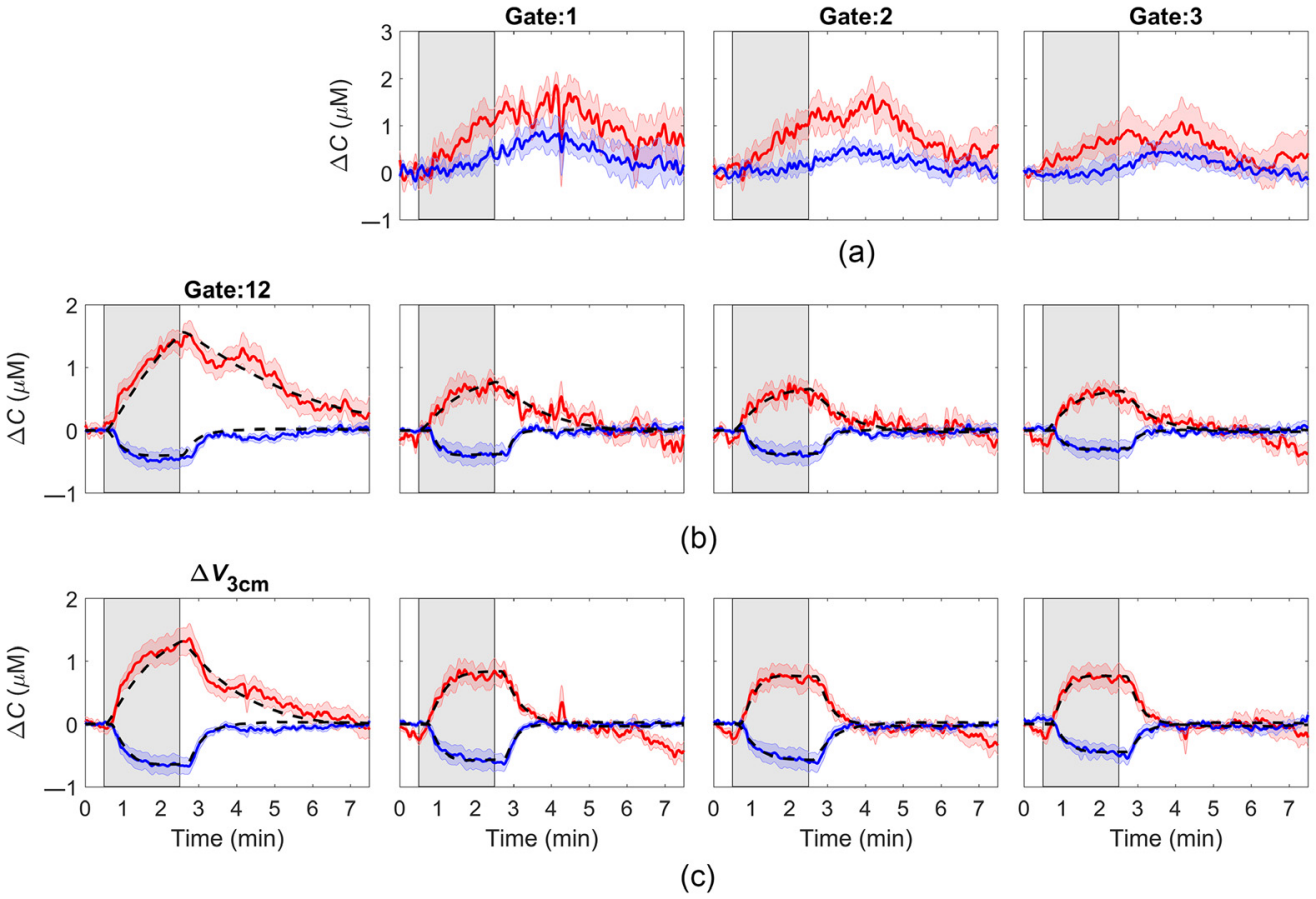
Average $\Delta C_{\mathrm{HbO}}$ (red) and $\Delta C_{\mathrm{Hb}}$ (blue) responses to a 2 min hypercapnic challenge (indicated by the shaded gray region) from data recorded at $r_{\mathrm{SD}}=3\;\mathrm{cm}$. (a) Responses from the first gates (gate # 1–3); (b) responses from the last gate (gate # 12) before and after applying regression using one of gates 1 to 3 as the regressor; and (c) responses derived from the variance signal before and after applying the same regression. All time courses were averaged across nine subjects, and shading surrounding each line represents the standard error of the mean. The best fits of the hemodynamic model to average hemoglobin signals are illustrated in each graph by the black dashed line.

Figure 6 presents average $\Delta C_{\mathrm{HbO}}$ and $\Delta C_{\mathrm{Hb}}$ responses to hypercapnia derived from the first three gates for DTOFs recorded at $r_{\mathrm{SD}}=3\;\mathrm{cm}$ [Fig. 6(a)]. Similar to the times series derived from $\Delta N_{1\;\mathrm{cm}}$ (Fig. 5(a)), all time series exhibited noticeable residue signals after $P_{\mathrm{ET}}{\mathrm{CO}}_{2}$ had returned to normocapnia, particularly for $\Delta C_{\mathrm{HbO}}$. These residues were also evident in the $\Delta C_{\mathrm{HbO}}$ time courses derived from gate 12 at $r_{\mathrm{SD}}=3\;\mathrm{cm}$ [Fig. 6(b)] and, to a lesser extent, from $\Delta V_{3\;\mathrm{cm}}$ [Fig. 6(c)]. Similar to Fig. 5, $\Delta C_{\mathrm{HbO}}$ and $\Delta C_{\mathrm{Hb}}$ time courses obtained after regression analysis using an early gate as the regressor exhibited improved dynamics of the reconstructed hypercapnic responses for both gate 12 and $\Delta V_{3\;\mathrm{cm}}$. No significant difference was found in the goodness of fit using an early gate as the regressor compared to using $\Delta N_{1\;\mathrm{cm}}$ as the regressor.

Table 2 provides the best-fit estimates of τ and ssCVR from the analysis of $\Delta C_{\mathrm{HbO}}$ and $\Delta C_{\mathrm{Hb}}$ time courses obtained with an early gate signal ($r_{\mathrm{SD}}=3\;\mathrm{cm}$) as the regressor and either gate 12 or $\Delta V_{3\;\mathrm{cm}}$ as the dependent variable. The ANOVA indicated that τ and ssCVR estimates for both dependent variables were independent of which early gate was chosen as the regressor. Table 2 includes the average τ and ssCVR values across the three early gates.

**Table 2:** Best fit estimates of the time constant (τ) and steady-state cerebrovascular reactivity (ssCVR) obtained from the analysis of the $\Delta C_{\mathrm{HbO}}$, $\Delta C_{\mathrm{Hb}}$ data derived from the regression approach based on combinations of early and late gates and by combining $\Delta V_{3\;\mathrm{cm}}$ and an early gate. Values are presented as average (standard deviation) across subjects.

	Regressor	τ (s)		ssCVR (μM/mmHg)	
Oxyhemoglobin (HbO)		The late gate (gate no. 12)	$\Delta V_{3\;\mathrm{cm}}$	The late gate (gate no. 12)	$\Delta V_{3\;\mathrm{cm}}$
	—	101±51	77±45	0.12±0.03	0.09±0.02
	Gate 1	33±22^a^	20±18^a^	0.06±0.04^a^	0.06±0.03^a^
	Gate 2	41±70	20±19^a^	0.06±0.05^a^	0.06±0.03^a^
	Gate 3	37±43^a^	19±19^a^	0.07±0.05^a^	0.06±0.03^a^
	Average (gates 1 to 3)	37±50^a^	20±18^a^	0.06±0.05^a^	0.06±0.03^a^
Deoxyhemoglobin (Hb)		The late gate (gate no. 12)	$\Delta V_{3\;\mathrm{cm}}$	The late gate (gate no. 12)	$\Delta V_{3\;\mathrm{cm}}$
	—	33±34	31±13	−0.03±0.03	−0.05±0.03
	Gate 1	22±31	35±32	−0.03±0.03	−0.04±0.03
	Gate 2	28±42	35±36	−0.03±0.03	−0.04±0.03
	Gate 3	15±11	22±29	−0.03±0.03	−0.04±0.04
	Average (gates 1 to 3)	22±31	31±33	−0.03±0.03	−0.04±0.03

a Statistically significant differences between parameters recovered with and without regression analysis

**Fig. 7:**
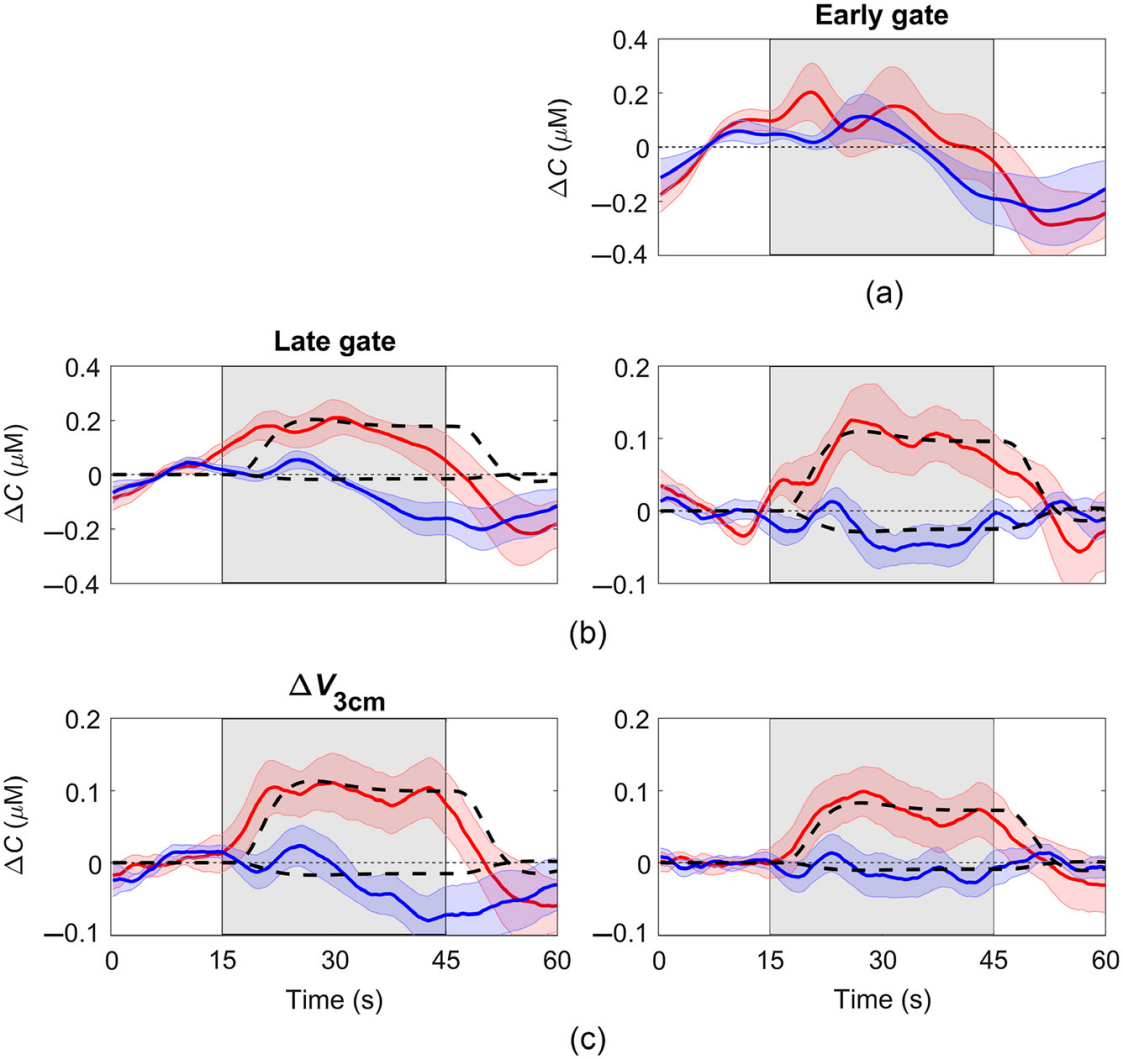
(a) Average $\Delta C_{\mathrm{HbO}}$ (red) and $\Delta C_{\mathrm{Hb}}$ (blue) response to functional activation (indicated by the shaded region) obtained for the early gate (mean of signal for gate # 2–3). (b) Regression analysis results obtained for the early gate (used as regressor) and the late gate (gate # 10). (c) Regression analysis results obtained for the early gate (used as regressor) and $\Delta V_{3\;\mathrm{cm}}$. Shading surrounding each line represents the standard error of the mean. The best fit of the hemodynamic model to each average hemoglobin time course is illustrated by the black dashed line.

Figure 7 shows the average $\Delta C_{\mathrm{HbO}}$ and $\Delta C_{\mathrm{Hb}}$ time courses obtained from the functional data for early and late gates, as well as from the variance signal [Fig. 7(a)]. Similar to the hypercapnia data, differences in hemoglobin time courses can be observed for responses obtained for signals primarily sensitive to the ECL (i.e., early gate) and signals with greater depth sensitivity (i.e., late gate or $\Delta V_{3\;\mathrm{cm}}$). In particular, the averaged hemoglobin time courses from the early gate exhibited a slow component that propagated throughout the 60 s period. The influence of this physiological signal was smaller but still observable in the $\Delta C_{\mathrm{HbO}}$ and $\Delta C_{\mathrm{Hb}}$ time series derived from the late gate or $\Delta V_{3\;\mathrm{cm}}$. Regression analysis using the early gate signal as the regressor further reduced the effect of the ECL component, resulting in $\Delta C_{\mathrm{HbO}}$ and $\Delta C_{\mathrm{Hb}}$ time courses that were similar to time course predicted by the HRF. This was confirmed by the reduction of the values of the $\chi^{2}$ goodness of fit obtained for late gate or $\Delta V_{3\;\mathrm{cm}}$ after applying regression analysis; however, statistical significance was only achieved for $\Delta C_{\mathrm{Hb}}$ calculated using $\Delta V_{3\;\mathrm{cm}}$.

## 4 Discussion

The main challenge with fNIRS studies involving adults is the substantial signal contamination from the ECL. By isolating late-arriving photons, trNIRS provides the ability to fundamentally improve depth sensitivity. This advantage is demonstrated by the temporal differences in the reconstructed hemoglobin time courses shown in Fig. 5(a). The magnitude of the residue signal following the hypercapnic challenge (i.e., after $P_{\mathrm{ET}}{\mathrm{CO}}_{2}$ returned to normocapnia) diminished as the order of the statistical moment increased. This trend was more pronounced for $\Delta C_{\mathrm{HbO}}$ due to its greater sensitivity to scalp hemodynamics.^20^ However, higher moments and late gates are still sensitive to the ECL, as indicated by the sensitivity factors shown in Figs. 2 and 3. Consequently, the time constant τ, which characterizes the dynamics of cerebrovascular reactivity was larger than expected for $\Delta C_{\mathrm{HbO}}$ determined from $\Delta V_{3\;\mathrm{cm}}$ (τ=77±45  s) compared to functional magnetic resonance imaging studies (fMRI).^33^^,^^35^ For healthy gray matter, τ should be in the range of 15 to 40 s, as illustrated by the theoretical response shown in Fig. 5(b).

This study focused on investigating if the depth sensitivity of trNIRS could be further improved by incorporating regression analysis. To emulate the method proposed by Saager and Berger^6^ for CW NIRS applications that include a short-separation channel, regression was first applied to trNIRS data recorded at $r_{\mathrm{SD}}=3\;\mathrm{cm}$ using the $\Delta N_{1\;\mathrm{cm}}$ as the regressor. The improvement to the hypercapnic response was demonstrated by the reduced residue signal observed in the recovery phase [Fig. 5(b)]. Again, the benefit was greater for $\Delta C_{\mathrm{HbO}}$ as indicated by the significant reduction in τ for the time series obtained from $\Delta {\left\langle t\right\rangle }_{3\;\mathrm{cm}}$ and $\Delta V_{3\;\mathrm{cm}}$ (Table 1). The corresponding τ values for $\Delta C_{\mathrm{Hb}}$ from all three moments were also lower after regression; however, only the results for $\Delta N_{3\;\mathrm{cm}}$ reached statistical significance. In agreement with CW NIRS studies, these results confirm that applying regression to trNIRS data that includes a short-separation channel is beneficial, even for trNIRS metrics with enhanced depth sensitivity.

A further advantage of trNIRS is the possibility to apply a regression to data acquired at a single $r_{\mathrm{SD}}$ since early arriving photons should be predominately sensitive to the ECL. This is reflected in the sensitivity factors shown in Fig. 4(a) for the first three gates that span the initial rise of a typical DTOF. The contribution from the brain is expected to be <3% for all three gates assuming the distance to the brain is 14 mm.^20^ To evaluate the feasibility of using an early gate as the regressor, this study focused on the two metrics that provided the greatest depth sensitivity (the variance and the 250-ps gate positioned at the end of the DTOF, both measured at $r_{\mathrm{SD}}=3\;\mathrm{cm}$) to act as the dependent variable in the regression analysis. In agreement with the results involving the short-separation channel, applying regression to signals obtained from the same DTOF substantially reduced the residue signal in the hypercapnia experiments [Figs. 6(b)–6(c)]. For both the late gate and $\Delta V_{3\;\mathrm{cm}}$, τ for $\Delta C_{\mathrm{HbO}}$ was significantly lower compared to the values derived from each metric on its own, and the regression also reduced inter-subject variability (Table 2). Regression appeared to work better for $\Delta V_{3\;\mathrm{cm}}$ compared to the late gate (i.e., τ=20±18  s versus 37±50  s) likely due to the greater depth sensitivity of the former, but this difference did not reach statistical significance. Regression did not significantly improve the time constant for the corresponding $\Delta C_{\mathrm{Hb}}$ time courses, which likely reflects the lower residue signal compared to $\Delta C_{\mathrm{HbO}}$. In general, the average τ values following regression for both hemoglobin signals were within the expected range reported in fMRI studies.^33^^,^^35^

No significant differences were found between fitting parameter estimates (τ and ssCVR) for regression involving the three early gates, suggesting that any of the gates, or some combination, would be adequate to act as the regressor. The lack of any difference indicates that selection of the time window for the early gate is not overly critical, provided it is predominately sensitive to the ECL. There may be incidences in which it would be prudent to select the earliest gate possible due to variations in ECL thickness between individuals and across the head.^36^ In this study, the average $\Delta C_{\mathrm{HbO}}$ and $\Delta C_{\mathrm{Hb}}$ time courses from gate 1 appeared to exhibit greater variability compared to those generated from gates 2 and 3 [Fig. 6(a)]. This may have been caused by light contamination related to differences in probe-to-skin contact but it did not influence the overall fitting results. Interestingly, the average $\Delta C_{\mathrm{HbO}}$ and $\Delta C_{\mathrm{Hb}}$ time courses extracted from the early gates were not the same as those obtained from $\Delta N_{1\;\mathrm{cm}}$. All three $\Delta C_{\mathrm{Hb}}$ time courses from the early gates at $r_{\mathrm{SD}}=3\;\mathrm{cm}$ increased in response to hypercapnia, whereas the corresponding $\Delta N_{1\;\mathrm{cm}}$ decreased during the same period. One possible explanation is that $\Delta N_{1\;\mathrm{cm}}$ contained some brain signal considering the corresponding average $\Delta C_{\mathrm{Hb}}$ time course exhibited a decrease near the onset of hypercapnia similar to that observed in the time courses with greater sensitivity to the brain. To investigate this possibility, time series were extracted for the first 250 ps of the $\Delta N_{1\;\mathrm{cm}}$ data, and these resulted in similar $\Delta C_{\mathrm{HbO}}$ and $\Delta C_{\mathrm{Hb}}$ time courses as shown in Fig. 5(a) (data not presented). A most likely explanation would be regional variations in scalp hemodynamics that led to different deoxyhemoglobin contributions measured by the two detectors.

The regression approach was also applied to previously acquired fNIRS data involving a motor imagery task. The average $\Delta C_{\mathrm{HbO}}$ and $\Delta C_{\mathrm{Hb}}$ time courses were improved by applying the regression approach as reflected by the removal of a slow frequency component observed in Figure 6 and the improved $\chi^{2}$ goodness of fit between the experimental data and the GLM, although this improvement did not reach statistical significance. One of the challenges with assessing the performance of the regression approach on activation data is that the magnitude of scalp signal changes likely varies considerably between subjects. This is in contrast to the hypercapnic challenge that was both global (i.e., can cause hemodynamic responses in multiple tissues including scalp) and fairly robust across participants due to the relatively large increase in $P_{\mathrm{ET}}{\mathrm{CO}}_{2}$ (14.2±0.6  mmHg).^20^ It would be useful to apply the regression approach to larger functional trNIRS data sets to further confirm its value for improving the sensitivity to the brain. Lastly, this study implemented the linear least-squares method first proposed by Saager and Berger, and the technique would likely benefit from refinements such as incorporating adaptive filters.^3^

## 5 Conclusions

In summary, this study demonstrated that applying regression analysis to trNIRS metrics with different depth sensitivities improved the characterization of dynamic cerebrovascular reactivity and the oxygenation responses during an activation task. The unique ability of trNIRS to extract the necessary information regarding the ECL from the same data set used to detect changes in cerebral oxygenation ensures the regressor truly reflects the ECL contribution to each probe. The challenge with trNIRS is the additional cost and complexity of the equipment—in this study, all measurements were acquired with a four-channel system. However, this bottleneck is likely to be overcome with the development of miniaturized laser sources and compact silicon photomultiplier devices,^16^^,^^37^^,^^38^ which will enable multi-channel trNIRS systems to rival current CW NIRS devices.

## References

[1] Ferrari M. Quaresima V., “A brief review on the history of human functional near-infrared spectroscopy (fNIRS) development and fields of application,” Neuroimage 63(2), 921–935 (2012). NEIMEF 1053-8119 10.1016/j.neuroimage.2012.03.049 22510258

[r2] Chen W. L. et al., “Functional near-infrared spectroscopy and its clinical application in the field of neuroscience: advances and future directions,” Front. Neurosci. 14, 724 (2020).1662-453X 10.3389/fnins.2020.00724 32742257 PMC7364176

[3] Scholkmann F. et al., “A review on continuous wave functional near-infrared spectroscopy and imaging instrumentation and methodology,” Neuroimage 85, 6–27 (2014). NEIMEF 1053-8119 10.1016/j.neuroimage.2013.05.004 23684868

[4] Yücel M. A. et al., “Mayer waves reduce the accuracy of estimated hemodynamic response functions in functional near-infrared spectroscopy,” Biomed. Opt. Express 7(8), 3078–3088 (2016). BOEICL 2156-7085 10.1364/BOE.7.003078 27570699 PMC4986815

[5] Gagnon L. et al., “Quantification of the cortical contribution to the NIRS signal over the motor cortex using concurrent NIRS-fMRI measurements,” Neuroimage 59(4), 3933–3940 (2012). NEIMEF 1053-8119 10.1016/j.neuroimage.2011.10.054 22036999 PMC3279595

[6] Saager R. B. Berger A. J., “Direct characterization and removal of interfering absorption trends in two-layer turbid media,” J. Opt. Soc. Am. A 22(9), 1874 (2005). JOAOD6 0740-3232 10.1364/JOSAA.22.001874 16211814

[r7] Saager R. Berger A., “Measurement of layer-like hemodynamic trends in scalp and cortex: implications for physiological baseline suppression in functional near-infrared spectroscopy,” J. Biomed. Opt. 13(3), 034017 (2008). JBOPFO 1083-3668 10.1117/1.2940587 18601562

[8] Gagnon L. et al., “Short separation channel location impacts the performance of short channel regression in NIRS,” Neuroimage 59(3), 2518–2528 (2012). NEIMEF 1053-8119 10.1016/j.neuroimage.2011.08.095 21945793 PMC3254723

[9] Milej D. et al., “Direct assessment of extracerebral signal contamination on optical measurements of cerebral blood flow, oxygenation, and metabolism,” Neurophotonics 7(4), 045002 (2020). 10.1117/1.NPh.7.4.045002 33062801 PMC7540337

[10] Liebert A. et al., “Evaluation of optical properties of highly scattering media by moments of distributions of times of flight of photons,” Appl. Opt. 42(28), 5785 (2003). APOPAI 0003-6935 10.1364/AO.42.005785 14528944

[11] Milej D. et al., “Time-resolved multi-channel optical system for assessment of brain oxygenation and perfusion by monitoring of diffuse reflectance and fluorescence,” Opto-Electron. Rev. 22(1), 55–67 (2014). OELREM 1230-3402 10.2478/s11772-014-0178-y

[12] Selb J. Joseph D. K. Boas D. A., “Time-gated optical system for depth-resolved functional brain imaging,” J. Biomed. Opt. 11(4), 044008 (2006). JBOPFO 1083-3668 10.1117/1.2337320 16965165

[13] Zucchelli L. et al., “Method for the discrimination of superficial and deep absorption variations by time domain fNIRS,” Biomed. Opt. Express 4(12), 2893–2910 (2013). BOEICL 2156-7085 10.1364/BOE.4.002893 24409389 PMC3862167

[r14] Milej D. et al., “Optimization of the method for assessment of brain perfusion in humans using contrast-enhanced reflectometry: multidistance time-resolved measurements,” J. Biomed. Opt. 20(10), 106013 (2015). JBOPFO 1083-3668 10.1117/1.JBO.20.10.106013 26509415

[r15] Abdalmalak A. et al., “Can time-resolved NIRS provide the sensitivity to detect brain activity during motor imagery consistently?” Biomed. Opt. Express 8(4), 2162 (2017). BOEICL 2156-7085 10.1364/BOE.8.002162 28736662 PMC5516814

[r16] Lange F. Tachtsidis I., “Clinical brain monitoring with time domain NIRS: a review and future perspectives,” Appl. Sci. 9(8), 1612 (2019). 10.3390/app9081612

[17] Torricelli A. et al., “Time domain functional NIRS imaging for human brain mapping,” Neuroimage 85(Pt. 1), 28–50 (2014). NEIMEF 1053-8119 10.1016/j.neuroimage.2013.05.106 23747285

[18] Liebert A. et al., “Bed-side assessment of cerebral perfusion in stroke patients based on optical monitoring of a dye bolus by time-resolved diffuse reflectance,” Neuroimage 24(2), 426–435 (2005). NEIMEF 1053-8119 10.1016/j.neuroimage.2004.08.046 15627584

[19] Wabnitz H. et al., “Depth-selective data analysis for time-domain fNIRS: moments vs. time windows,” Biomed. Opt. Express 11(8), 4224–4243 (2020). BOEICL 2156-7085 10.1364/BOE.396585 32923038 PMC7449728

[20] Milej D. et al., “Characterizing dynamic cerebral vascular reactivity using a hybrid system combining time-resolved near-infrared and diffuse correlation spectroscopy,” Biomed. Opt. Express 11(8), 4571 (2020). BOEICL 2156-7085 10.1364/BOE.392113 32923065 PMC7449704

[21] Milej D. et al., “Time-resolved subtraction method for measuring optical properties of turbid media,” Appl. Opt. 55(7), 1507 (2016). APOPAI 0003-6935 10.1364/AO.55.001507 26974605

[22] Milej D. et al., “Subtraction-based approach for enhancing the depth sensitivity of time-resolved NIRS,” Biomed. Opt. Express 7(11), 4514 (2016). BOEICL 2156-7085 10.1364/BOE.7.004514 27895992 PMC5119592

[23] Zhang Q. Strangman G. E. Ganis G., “Adaptive filtering to reduce global interference in non-invasive NIRS measures of brain activation: how well and when does it work?” Neuroimage 45(3), 788–794 (2009). NEIMEF 1053-8119 10.1016/j.neuroimage.2008.12.048 19166945 PMC2671198

[24] Abdalmalak A. et al., “Assessing time-resolved fNIRS for brain-computer interface applications of mental communication,” Front. Neurosci. 14, 105 (2020).1662-453X 10.3389/fnins.2020.00105 32132894 PMC7040089

[25] Milej D. et al., “Quantification of cerebral blood flow in adults by contrast-enhanced near-infrared spectroscopy: validation against MRI,” J. Cereb. Blood Flow Metab. 40(8), 1672–1684 (2020). 10.1177/0271678X19872564 31500522 PMC7370369

[r26] Kewin M. et al., “Evaluation of hyperspectral NIRS for quantitative measurements of tissue oxygen saturation by comparison to time-resolved NIRS,” Biomed. Opt. Express 10(9), 4789 (2019). BOEICL 2156-7085 10.1364/BOE.10.004789 31565525 PMC6757477

[27] Abdalmalak A. et al., “Using fMRI to investigate the potential cause of inverse oxygenation reported in fNIRS studies of motor imagery,” Neurosci. Lett. 714, 134607 (2020). NELED5 0304-3940 10.1016/j.neulet.2019.134607 31693928

[28] Milej D. et al., “Quantification of blood-brain barrier permeability by dynamic contrast-enhanced NIRS,” Sci. Rep. 7(1), 1702 (2017). SRCEC3 2045-2322 10.1038/s41598-017-01922-x 28490806 PMC5431887

[29] Re R. et al., “Multi-channel medical device for time domain functional near infrared spectroscopy based on wavelength space multiplexing,” Biomed. Opt. Express 4(10), 2231–2246 (2013). BOEICL 2156-7085 10.1364/BOE.4.002231 24156079 PMC3799681

[30] Patterson M. S. Chance B. Wilson B. C., “Time resolved reflectance and transmittance for the non-invasive measurement of tissue optical properties,” Appl. Opt. 28(12), 2331–2336 (1989). APOPAI 0003-6935 10.1364/AO.28.002331 20555520

[31] Kacprzak M. et al., “Application of a time-resolved optical brain imager for monitoring cerebral oxygenation during carotid surgery,” J. Biomed. Opt. 17(1), 016002 (2012). JBOPFO 1083-3668 10.1117/1.JBO.17.1.016002 22352652

[32] Gerega A. et al., “Multiwavelength time-resolved near-infrared spectroscopy of the adult head: assessment of intracerebral and extracerebral absorption changes,” Biomed. Opt. Express 9(7), 2974 (2018). BOEICL 2156-7085 10.1364/BOE.9.002974 29984079 PMC6033559

[33] Poublanc J. et al., “Measuring cerebrovascular reactivity: the dynamic response to a step hypercapnic stimulus,” J. Cereb. Blood Flow Metab. 35(11), 1746–1756 (2015). 10.1038/jcbfm.2015.114 26126862 PMC4635229

[34] McKetton L. et al., “The aging brain and cerebrovascular reactivity,” Neuroimage 181, 132–141 (2018). NEIMEF 1053-8119 10.1016/j.neuroimage.2018.07.007 29981482

[35] Duffin J. et al., “The dynamics of cerebrovascular reactivity shown with transfer function analysis,” Neuroimage 114, 207–216 (2015). NEIMEF 1053-8119 10.1016/j.neuroimage.2015.04.029 25891374

[36] Strangman G. E. Zhang Q. Li Z., “Scalp and skull influence on near infrared photon propagation in the Colin27 brain template,” Neuroimage 85, 136–149 (2014). NEIMEF 1053-8119 10.1016/j.neuroimage.2013.04.090 23660029

[37] Di Sieno L. et al., “Miniaturized pulsed laser source for time-domain diffuse optics routes to wearable devices,” J. Biomed. Opt. 22(8), 085004 (2017). 10.1117/1.JBO.22.8.085004 28823112

[38] Saha S. et al., “Wearable SiPM-based NIRS interface integrated with pulsed laser source,” IEEE Trans. Biomed. Circuits Syst. 13, 1313–1323 (2019). 10.1109/TBCAS.2019.2951539 31689208

